# Clinical Evaluation of a Multi‐Component Facial Mask for Moisturizing, Repairing, and Anti‐Aging Effects

**DOI:** 10.1111/jocd.70355

**Published:** 2025-08-06

**Authors:** Fan Yang, Miao Guo, Jinjin Zhu, Hua Wang

**Affiliations:** ^1^ Department of Biotechnology, College of Life Science and Technology Huazhong University of Science and Technology Wuhan China; ^2^ Research & Development Center Mageline Biology Tech Co. Ltd. Wuhan Hubei China; ^3^ Department of Dermatology, Union Hospital, Tongji Medical College Huazhong University of Science and Technology (HUST) Wuhan China

**Keywords:** facial mask, moisturization, skin aging, skin barrier, wrinkles

## Abstract

**Background:**

Facial masks are commonly used in skincare for targeted and intensive treatment. However, masks that provide only moisturizing benefits have limited functionality and may not adequately meet comprehensive anti‐aging needs.

**Objectives:**

To design and evaluate the efficacy of a multi‐component facial mask for moisturizing, repairing, and anti‐aging effects.

**Methods:**

The clinical efficacy of the facial mask was evaluated in a 28‐day study, with skin parameters measured immediately after application, and at 14 and 28 days. Skin hydration and transepidermal water loss (TEWL) were measured using a Corneometer and a Vapometer, respectively. Skin elasticity and firmness were assessed using a Cutometer. Facial photographs were captured using the VISIA CR system, and wrinkles and roughness were analyzed with Antera 3D.

**Results:**

Immediate and 28‐day assessments revealed significant improvements in skin hydration and reduced TEWL (*p* < 0.001). After 28‐day use, skin elasticity and firmness increased by 17.31% and 16.18%, respectively. VISIA CR analysis demonstrated enhanced skin gloss and decreased redness. Antera 3D measurements indicated significant reductions in the length and width of under‐eye wrinkles and crow's feet, along with improved skin texture (*p* < 0.001). Participant self‐assessments reported high satisfaction with the mask's safety and efficacy, both in the short term and long term.

**Conclusion:**

The multi‐component facial mask demonstrated significant efficacy in enhancing skin hydration, improving barrier repair, and providing anti‐aging benefits.

## 1 Introduction

Skin aging is a multifaceted process influenced by intrinsic factors, such as genetic predispositions, and extrinsic factors, including environmental exposures and lifestyle choices [1, 2, 3]. As the skin ages, it exhibits visible changes, including reduced hydration, diminished elasticity, increased skin roughness, and wrinkle formation [4, 5, 6]. These manifestations result from complex biological processes affecting skin structure and function, including the degradation of extracellular matrix components, reduced collagen and elastin production, and impaired skin barrier function [7, 8].

In recent years, there has been a growing interest in the development of cosmetic interventions, such as serums, creams, and masks, aimed at addressing the signs of aging [9]. Among these, facial masks have gained popularity due to their ability to deliver targeted benefits, intensive treatments, and enhance the penetration of active ingredients [10, 11]. These masks are well‐regarded for providing immediate hydration and improving skin texture [12, 13]. However, many facial masks provide only short‐term benefits, primarily focusing on hydration while lacking the advanced anti‐aging properties necessary for long‐term skin repair and rejuvenation [14, 15]. An effective anti‐aging facial mask should incorporate a range of ingredients specifically designed to deliver multiple benefits, including moisturizing, barrier repair, and anti‐aging effects.

Yeast/rice fermentation filtrate (RFF), a naturally sourced fermentative ingredient abundant in polysaccharides, amino acids, organic acids, and minerals, has attracted attention for its superior moisturizing and skin‐repairing benefits [16, 17]. Moisturizing agents, such as hydrolyzed sodium hyaluronate (HyHA) and acetylated sodium hyaluronate (AcHA), are well‐known skincare ingredients due to their deep hydration properties, which enhance skin plumpness and reduce the appearance of wrinkles [18]. Additionally, hydroxypropyl tetrahydropyrantriol, also known as C‐xyloside, has been shown to enhance the synthesis of glycosaminoglycans and proteoglycans in the skin, promote the production of collagen types IV and VII, and strengthen the epidermal‐dermal junction [19, 20]. N‐acetylglucosamine has also been shown to enhance skin hydration, stimulate hyaluronic acid production, and promote collagen synthesis, thereby reducing fine lines and improving skin texture [21].

Signaling peptides play a crucial role in skin rejuvenation [22]. Specifically, Palmitoyl Tripeptide‐5 promotes collagen synthesis while inhibiting its degradation, resulting in reduced wrinkles, enhanced skin elasticity, and improved skin firmness [23]. Moreover, biomimetic peptides, such as arginine/lysine polypeptide, provide immediate wrinkle reduction by blocking nerve signal transmission, mimicking the effects of botulinum toxin [24].

*Portulaca oleracea* extract, rich in flavonoids and polyphenols, neutralizes free radicals and reduces oxidative stress, while also exhibiting anti‐inflammatory effects by inhibiting inflammatory mediators such as IL‐6 and TNF‐α [25]. *Centella asiatica* extract promotes skin healing and collagen synthesis, effectively reducing redness and irritation by suppressing inflammatory signaling pathways, including NF‐κB [26]. Dipotassium glycyrrhizate (DG) significantly reduces skin inflammation by inhibiting the production of inflammatory mediators. It also enhances skin barrier function and promotes moisture retention [27]. Additionally, DG influences ion channels and regulates metabolic processes to alleviate discomfort. Hydroxyphenyl Propamidobenzoic Acid (HPA) is a compound similar to natural avenanthramides, a class of active compounds found in oats [28]. It promotes keratinocyte proliferation, migration, and tight junction protein expression, thereby strengthening the skin barrier.

In this study, we developed a versatile anti‐aging facial mask incorporating RFF, HyHA, AcHA, Palmitoyl Tripeptide‐5, arginine/lysine polypeptide, C‐xyloside, N‐acetylglucosamine, *Portulaca oleracea* extract, *Centella asiatica* extract, DG, and HPA. The sheet mask is made of soft, elastic fiber that stretches to conform to the face, ensuring a secure fit and optimal skin contact. In the clinical trial, participants applied the facial mask every 2 days for 28 days. The efficacy of the mask was evaluated through a combination of objective biophysical assessments and subjective participant evaluations.

## 2 Materials and Methods

### 2.1 Facial Mask Design and Formulation

The tested facial mask consisted of a face‐shaped elastic fiber sheet mask (~2 g) infused with mask essence (~32 g), supplied by Mageline Biology Tech Co. Ltd. The detailed formulation of the mask essence is provided in Table 1.

**TABLE 1:** Composition of the mask essence ingredients.

Functionality	Ingredients (INCI name)
Base ingredients	Water, dimethicone, squalane *Sesamum Indicum* (sesame) seed oil *Limnanthes Alba* (meadowfoam) seed oil Sucrose stearate, sodium surfactin
Moisturizing ingredients	Glycerin, butylene glycol 1,2‐hexanediol, 1,2‐pentanediol Hydrolyzed sodium hyaluronate Acetylated sodium hyaluronate
Anti‐aging ingredients	Yeast/rice fermentation filtrate Hydroxypropyl tetrahydropyrantriol Acetyl glucosamine, panthenol Palmitoyl tripeptide‐5 Arginine/lysine polypeptide
Skin barrier repair ingredients	*Portulaca Oleracea* extract *Centella Asiatica* extract Hydroxyphenyl propamidobenzoic acid Dipotassium glycyrrhizate

### 2.2 In Vivo Clinical Trial

The clinical study was performed at the Shanghai WEIPU Testing Technology Group Co. Ltd. The research protocol of this clinical study (No. SHA01‐24070419‐JC‐01) was reviewed and approved by the Shanghai Ethics Committee for Clinical Research. Possible benefits, risks, and complications were thoroughly explained to the participants, who all provided written informed consent prior to enrollment. The study was designed as a single‐center, single‐blind clinical trial, where participants were unaware of the treatment they received to prevent their expectations from influencing the results. However, the research team conducting the assessments was not blinded. A before and after control approach was employed, incorporating objective measurements, subjective evaluations, and both localized and full‐face photographs.

#### 2.2.1 Subjects and Study Protocol

A total of 33 participants were enrolled, comprising 5 males and 28 females, aged 39–59 years, with a mean age of 48.6 ± 0.9 years. Inclusion criteria included: (1) Healthy individuals aged 30–55 years; (2) Participants with dry, loose facial skin lacking elasticity, prone to redness, and with compromised barrier function; (3) Participants with multiple fine lines on the face (e.g., around the eyes, forehead), static lines in the nasolabial fold area, sagging in the lower face, and fine lines or wrinkles around the eyes, corresponding to Grades 1–5 in the SKIN AGING ATLAS; (4) Participants capable of cooperating with the study and maintaining a regular lifestyle during the research period; (5) Participants who can read, understand, and voluntarily sign the informed consent form; (6) Participants who agree not to use any cosmetics, drugs, or supplements that could affect the study results during the trial; (7) Other relevant inclusion criteria.

Exclusion Criteria were as follows: (1) Participants with skin diseases at the test sites that could affect the study results; (2) Participants with a history of severe allergies; (3) Pregnant or breastfeeding women, or those planning to become pregnant during the study; (4) Participants with severe heart, liver, or kidney dysfunction, or significantly compromised immune function; (5) Participants with mental disorders, severe endocrine diseases, or those taking oral contraceptives; (6) Participants who have participated in clinical drug trials or other studies within the past 30 days, or have used systemic drugs affecting the study results within the past week; (7) Participants who have used cosmetic products, either orally or topically, that could impact the study results within the last 2 weeks; (8) Participants unable to cooperate with the study; (9) Participants deemed unsuitable by the investigator; (10) Other relevant exclusion criteria.

The clinical trial was conducted from July 16 to August 13, 2024, coinciding with the local summer season, during which high humidity reduced the need for additional moisturizers. During recruitment, all participants signed an informed consent form provided by the testing institution, which explicitly prohibited the use of unapproved skincare products, including moisturizers, throughout the trial period. The facial cleansers used by the subjects were standardized and uniformly provided by the testing institute. These were commercially available, amino acid‐based formulations, devoid of active ingredients or specialized claims that could potentially interfere with the noninvasive assessments. To monitor participants' adherence to the guidelines, regular check‐ins were conducted through self‐reported daily diaries and phone reminders provided every other day. Only participants who fully adhered to the protocol were included in the final analysis. These measures ensured that only the standardized facial cleanser and the test masks were used throughout the study.

All participants used the facial mask for 28 days, applying it every 2 days. The application procedure was as follows: participants first cleansed their facial skin, then gently unfolded the mask and placed it on their face, pressing lightly to ensure a proper fit. After 20 min, the mask was removed, and participants massaged their face until the remaining essence was fully absorbed. The essence was left on the skin without rinsing, and no additional skincare products were applied afterward.

Noninvasive efficacy measurements were performed immediately after mask removal on Day 0. For subsequent assessments on Days 14 and 28, measurements were conducted after a 12‐h overnight period following mask removal.

#### 2.2.2 Biophysical Skin Assessment

Measurements were conducted in a controlled environment with air conditioning (temperature: 21°C ± 1°C, relative humidity: 50% ± 10%) following a minimum acclimatization period of 30 min. Clinical skin parameters were assessed using various biophysical techniques immediately after facial mask application, as well as after 14 and 28 days of use. Skin hydration was measured with a Corneometer (Courage & Khazaka GmbH, CM 825, Germany). Skin elasticity (R2) and firmness (F4) were evaluated using a Cutometer (Courage & Khazaka GmbH, Germany). Transepidermal water loss (TEWL) was measured with a Vapometer (Delfin Technologies). In the test, measurements using the Corneometer, Cutometer, and Vapometer were taken on the cheek, specifically at the intersection of the horizontal line passing through the center of the eyeball and the center of the nose bridge. Each measurement was performed three times at different locations to ensure accuracy, and the average value was used for analysis.

#### 2.2.3 Photography

Before VISIA CR and Antera 3D measurements, participants were instructed to acclimate in the measurement room for at least 30 min (temperature: 21°C ± 1°C, relative humidity: 50% ± 10%). The VISIA CR was used to capture facial photographs and evaluate parameters including redness (*a*‐value), redness area ratio, gloss, and translucency. Antera 3D was employed to obtain localized photographs of the eye area and analyze parameters such as the length and width of under‐eye wrinkles, crow's feet, and skin roughness.

#### 2.2.4 Subject Self‐Assessment

Participants completed self‐assessment questionnaires on the efficacy, user experience, and comfort of the facial mask immediately after application, as well as after 14 and 28 days of use.

### 2.3 Statistical Analysis

Statistical analyses were conducted using SPSS (version 25.0, IBM). For normally distributed data, a paired sample *t*‐test was employed, while non‐normally distributed data were analyzed using the Wilcoxon signed‐rank test. The data were presented as standard error of the mean (SEM). All statistical tests were two‐tailed, with a significance level set at *α* = 0.05. Statistical differences were considered significant for *p* < 0.05.

## 3 Results

### 3.1 Skin Hydration Improvement With Facial Mask Use

**FIGURE 1:**
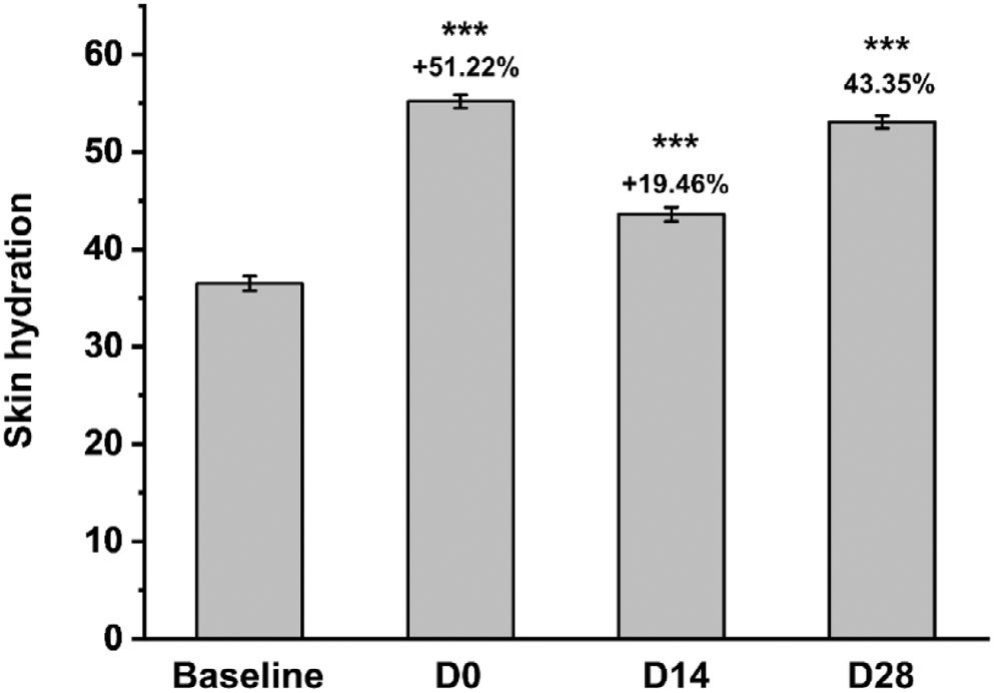
Variations in skin hydration before and after facial mask usage. ***p < 0.001 compared with the baseline.

A total of 33 subjects were screened, and ultimately, all subjects adhered to the protocol and were included in the final analysis. Figure 1 illustrates that a single application of the facial mask resulted in a significant increase in skin hydration by 51.22% (*p* < 0.001), indicating its potent moisturizing effect. After 14 days of continued use, skin hydration remained elevated with an improvement of 19.46%. Furthermore, following 28 days of consistent use, skin hydration increased notably by 43.35%.

### 3.2 Enhanced Skin Barrier Function

**FIGURE 2:**
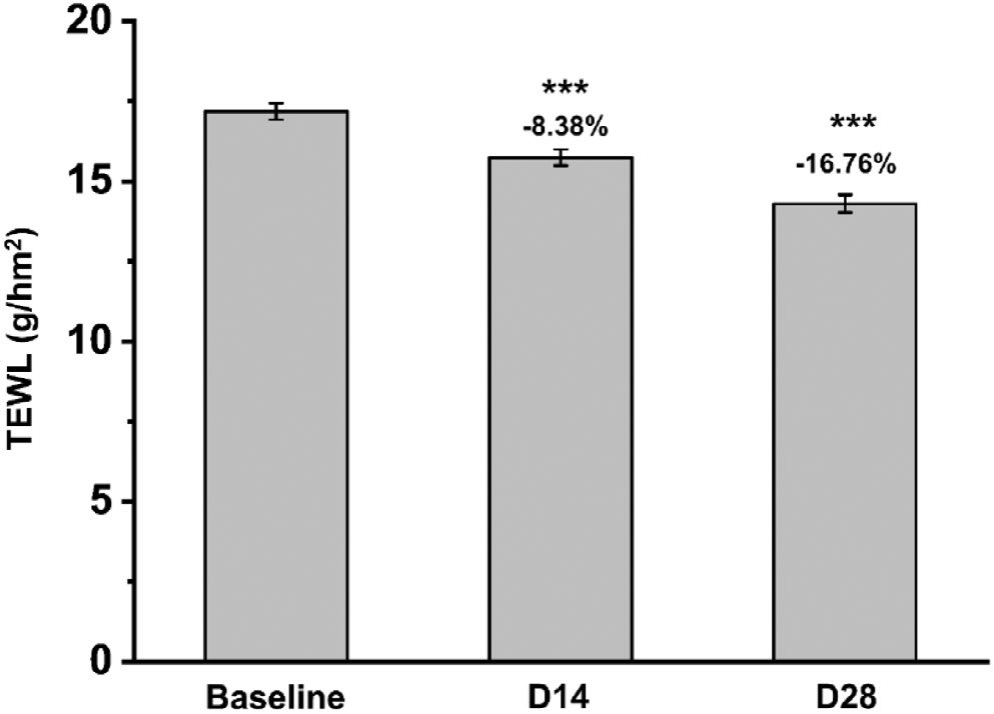
Variations in TWEL before and after facial mask usage. ***p < 0.001 compared with the baseline.

The significance of maintaining a healthy skin barrier is well‐established in dermatological research. In this study, TEWL was used as a measure of skin barrier function. After 14 days of facial mask application, TEWL decreased by 8.38% (*p* < 0.001), indicating a significant improvement in barrier function (Figure 2). Continued use for 28 days resulted in a 16.76% reduction in TEWL compared to baseline (*p* < 0.001), further demonstrating the mask's efficacy in enhancing skin barrier integrity.

### 3.3 Effects on Skin Elasticity and Firmness

**FIGURE 3:**
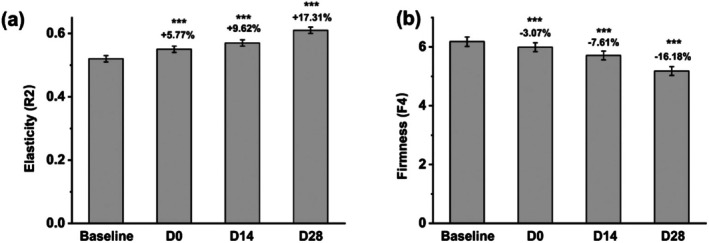
Variations in (a) skin elasticity and (b) firmness before and after facial mask usage. ***p < 0.001 compared with the baseline.

The effects of the facial mask on skin elasticity and firmness were evaluated immediately after application, as well as at 14 and 28 days. Figure 3a illustrates that skin elasticity increased by 5.77% immediately after application, by 9.62% at day 14, and by 17.31% at day 28. Similarly, skin firmness improved by 3.07% immediately after application, by 7.61% at day 14, and by 16.18% by day 28 (Figure 3b). These results demonstrate that the facial mask provides both immediate and sustained benefits in enhancing skin elasticity and firmness.

### 3.4 VISIA Results

**FIGURE 4:**
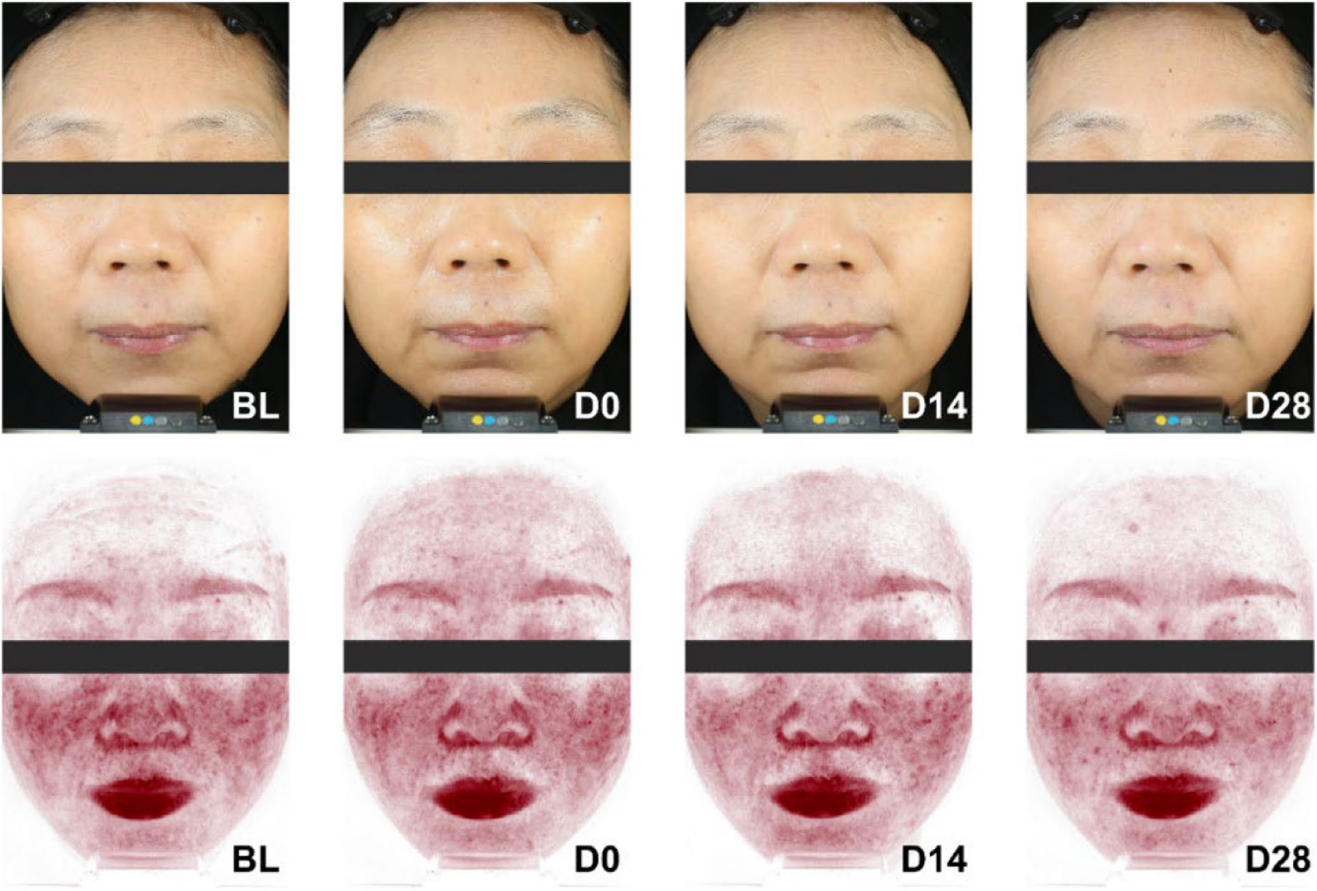
VISIA images taken by a volunteer in visible light and in red areas light source mode before and after facial mask usage for different time intervals.

**FIGURE 5:**
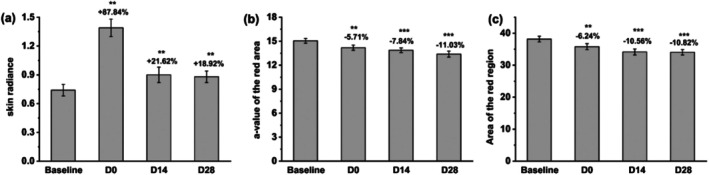
Variations in (a) skin radiance, (b) *a*‐value of the red area, and (c) area of the red region before and after facial mask usage. **p < 0.01; ***p < 0.001 compared with the baseline.

VISIA photo analysis demonstrated a significant enhancement in skin radiance, with an increase of 87.84% immediately after application, 21.62% at day 14, and 18.92% at day 28, as illustrated in Figures 4 and 5a. Additionally, Figure 4 shows the reduction in the red area observed through VISIA photos. The a‐value of the red area decreased by 5.71% immediately after application, 7.84% by day 14, and 11.03% by day 28. Similarly, the area of the red region decreased by 6.24% immediately after application, 10.56% by day 14, and 10.82% by day 28, as detailed in Figure 5b,c, respectively. These results highlight the mask's effectiveness in enhancing skin radiance and significantly reducing redness over the study period.

### 3.5 Antera 3D Results

**FIGURE 6:**
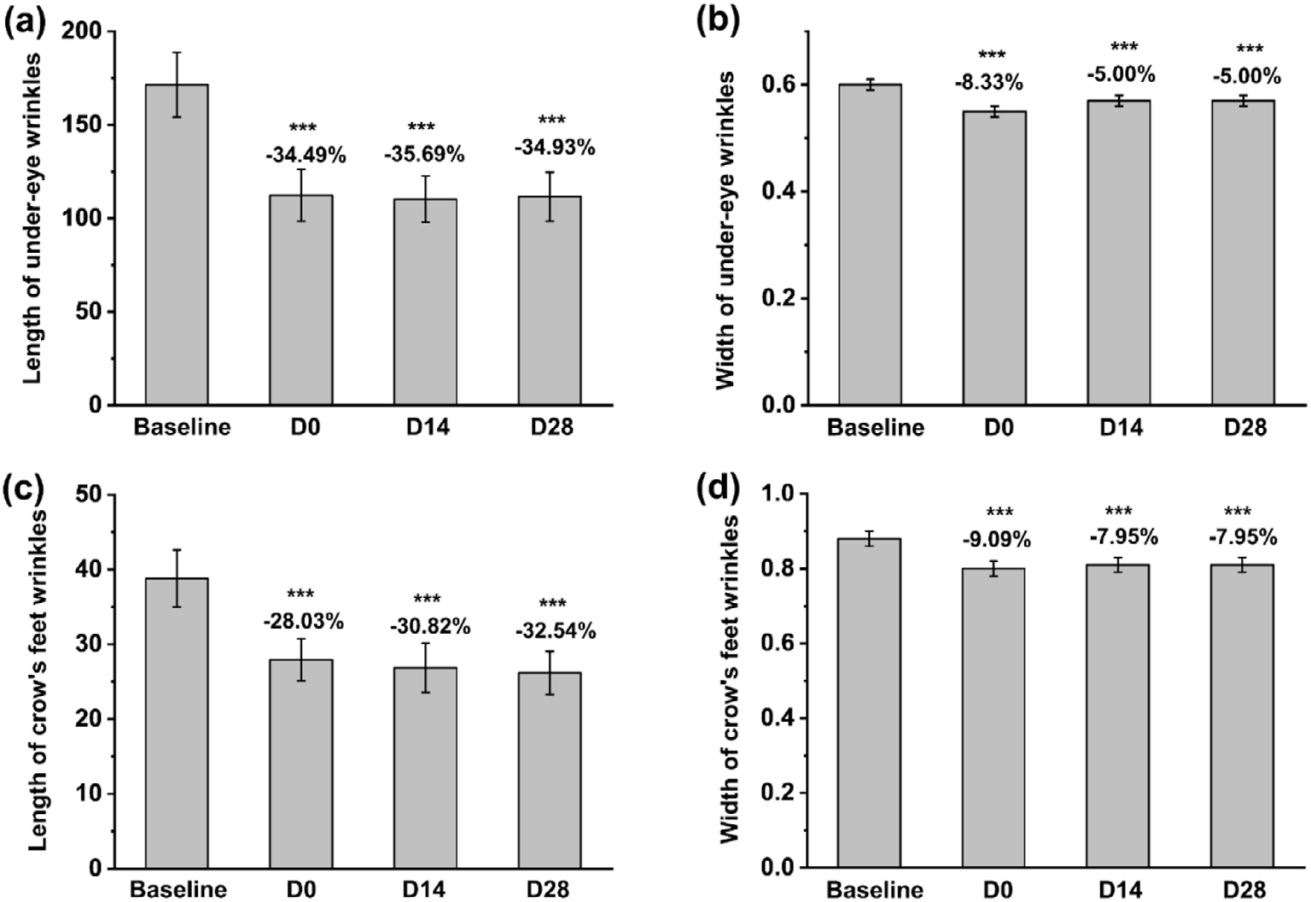
Statistical data of Antera 3D showing variations in (a) length of under‐eye wrinkles, (b) width of under‐eye wrinkles, (c) length of crow's feet wrinkles, and (d) width of crow's feet wrinkles before and after facial mask usage. ***p < 0.001 compared with the baseline.

Antera 3D analysis demonstrated significant improvements in wrinkle parameters following facial mask application. Immediately after application, the length of under‐eye wrinkles was reduced by 34.49%, with further reductions to 35.69% by day 14 and 34.93% by day 28 (Figure 6a). The width of under‐eye wrinkles decreased by 8.33% immediately after application, with further reductions of 5% at both day 14 and 28 (Figure 6b). The length of crow's feet wrinkles decreased by 28.03% immediately, 30.82% by day 14, and 32.54% by day 28, while the width decreased by 9.09% immediately and 7.95% at days 14 and 28 (Figure 6c,d).

**FIGURE 7:**
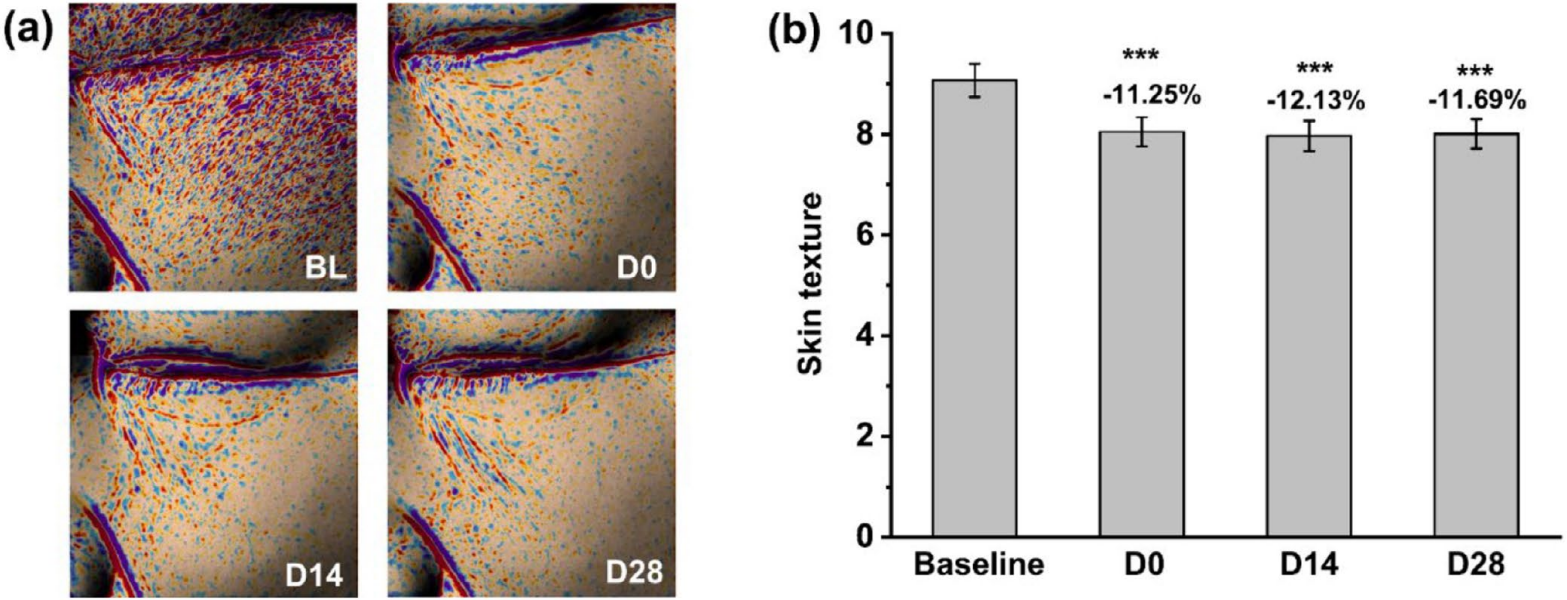
(a) Representative Antera 3D images illustrating improvements in skin texture before and after facial mask usage. (b) Statistical data of Antera 3D showing variations in skin textures. ***p < 0.001 compared with the baseline.

Representative Antera 3D images, shown in Figure 7a, illustrate the improvement in skin texture following the use of the facial mask. Statistical analysis, illustrated in Figure 7b, demonstrated significant improvements in skin texture, with an 11.25% increase immediately after application, a 12.13% increase at 14 days, and an 11.69% increase at 28 days, all of which were statistically significant.

### 3.6 Participant‐Reported Outcomes

The facial masks were well‐received, with 100% of participants reporting them as gentle and moisturizing at both 14 and 28 days (Table 2). Improvements were observed in skin brightness, texture, plumpness, and overall restorative effects, with these benefits becoming more pronounced over time. By day 28, all participants experienced enhanced elasticity, firmness, and a reduction in fine lines and crow's feet. Skin sensitivity and redness showed immediate improvement, with all participants reporting relief from discomfort by the end of the study. No adverse reactions, such as burning, tingling, or allergic responses, were reported throughout the study.

**TABLE 2:** Self‐assessment.

Question	D14	D28
Feel gentle and non‐irritating	100%	100%
Feel moisturizing after use	100%	100%
Feel brighter	94%	100%
Feel smoother in texture	97%	100%
Feel plumper	94%	97%
Feel restorative	94%	100%
Feel immediate improvement in skin sensitivity and redness after application	94%	100%
Feel soothing to skin discomfort after use	94%	97%
Feel more elastic and firmer after use	94%	100%
Feel reduction in fine lines after use	94%	100%
Feel improvement in crow's feet after use	91%	100%
Feel reduction in nasolabial folds after use	85%	100%
Feel improvement in frown lines after use	88%	94%
Feel reduction in forehead lines after use	91%	97%

## 4 Discussion

Skin aging is an inevitable process influenced by both intrinsic factors, such as genetics, and extrinsic factors, including ultraviolet (UV) radiation and environmental pollution [1]. This process manifests in visible signs such as reduced hydration, loss of elasticity, and the formation of wrinkles [4]. Facial masks have become a popular and effective skincare method due to their ability to deliver targeted ingredients and enhance the penetration of active compounds [12]. However, many facial masks primarily offer hydration, without offering skin rejuvenation benefits [14]. For instance, a recent study by Y. Wu et al. developed a moisturizing facial mask that showed efficacy only in improving skin barrier function and alleviating dryness [29]. These effects are insufficient to meet consumer expectations and the increasing demand for more comprehensive anti‐aging solutions.

In the present study, we designed a novel anti‐aging facial mask incorporating multiple active ingredients and evaluated its efficacy, safety, and tolerability in a randomized controlled trial. Our clinical findings demonstrate the mask's efficacy in enhancing skin hydration, restoring the skin barrier, improving texture, and reducing wrinkles. Skin hydration increased by 51.22% immediately and 43.35% after 28 days. A reduction in TEWL and alleviation of skin redness further highlight its barrier‐enhancing effects. Significant improvements in elasticity and firmness were observed after both single and 28‐day use, with a notable 87.84% increase in skin gloss, reflecting the mask's brightening effect. Wrinkle reduction in under‐eye and crow's feet areas was also significant, as shown by both single and 28‐day assessments. Antera 3D imaging confirmed improvements in skin texture. Consumer feedback supported these results, reporting moisturizing, repairing, soothing, brightening, and anti‐wrinkle benefits, with no adverse reactions. These findings affirm the mask's effectiveness and safety, making it a valuable addition to anti‐aging skincare routines.

The efficacy of the facial mask can be attributed to the synergistic effects of its active ingredients, which are specifically formulated for moisturizing, barrier repair, and anti‐aging. RFF, a naturally derived fermentative product rich in amino acids, polysaccharides, and vitamins, demonstrates excellent moisturizing and skin‐repairing properties [8]. Clinical studies have shown that RFF enhances skin barrier function, hydrates the skin, and reduces extrinsic photoaging, making it a promising ingredient for anti‐aging formulations [16]. HyHA and AcHA are highly effective moisturizers recognized for their deep hydration capabilities, which enhance skin plumpness and reduce the appearance of wrinkles [18]. C‐xyloside, a renowned anti‐aging ingredient, has been shown to enhance glycosaminoglycan synthesis and improve the ultrastructure of the dermal‐epidermal junction [20]. Numerous studies have demonstrated its clinical efficacy in reducing facial wrinkles, enhancing skin radiance, and improving skin firmness [19]. N‐acetylglucosamine improves skin hydration, stimulates hyaluronic acid production, and promotes collagen synthesis, thereby effectively reducing fine lines and enhancing skin smoothness [21]. Palmitoyl Tripeptide‐5 stimulates collagen synthesis and inhibits its degradation, leading to a reduction of wrinkles and an improvement in skin firmness. Arginine/lysine polypeptide provides rapid wrinkle reduction by blocking nerve signal transmission, similar to the effects of botulinum toxin [24].

Skin barrier dysfunction is a hallmark of sensitive skin and contributes to the onset of premature aging [4]. In our facial mask, *Portulaca oleracea* extract neutralizes free radicals, reduces oxidative stress, and inhibits inflammatory mediators such as IL‐6 and TNF‐α [25]. *Centella asiatica* extract promotes skin healing and alleviates redness and irritation by suppressing inflammatory signaling pathways, including NF‐κB [26]. DG reduces skin inflammation and enhances barrier function by inhibiting inflammatory mediators and regulating ion channels and metabolic processes [27]. HPA, also known as dihydroavenanthramide D, is a compound similar to natural avenanthramides derived from oats. It enhances keratinocyte proliferation, migration, and tight junction protein expression, thereby further strengthening the skin barrier [28]. These components work synergistically through various mechanisms to amplify each other's anti‐inflammatory effects, providing enhanced soothing and reparative benefits. This synergistic action results in a significant reduction in skin redness, with a 6.24% improvement after a single use and a 10.82% improvement after 28 days of continuous use.

The effectiveness and comfort of the facial mask can also be attributed to the unique materials and design of the mask sheet. Our mask features a soft, breathable, and elastic fiber sheet that ensures a comfortable fit for all participants. The elastic properties of the fiber allow the mask to stretch and conform to the contours of the face, providing a secure fit that maximizes skin contact. Moreover, this design enhances adherence, increases the surface area for optimal essence absorption, and prevents dripping, maintaining moisture throughout wear. The mask sheet synergizes with the essence, capable of holding up to 16 times its weight in essence. Its closed design, coupled with a unique ‘micro‐press’ structure, creates a sealed environment that significantly enhances the permeability of active ingredients. Due to the non‐greasy and moisturizing properties of the essence, the mask was removed after 20 min, leaving the essence on the skin without rinsing. This design allows the mask to function more like a long‐lasting anti‐aging serum, rather than a typical 20‐min mask. The extended contact time of the essence with the skin provides sustained benefits, thereby enhancing the overall efficacy of the facial mask.

Importantly, our facial mask is formulated to be gentle and non‐irritating, excluding commonly used preservatives such as sodium benzoate, potassium sorbate, and parabens [30]. Additionally, phenoxyethanol, a preservative frequently associated with irritation in sensitive skin, was deliberately avoided. Instead, we incorporated alternative preservatives, specifically 1,2‐hexanediol and 4‐hydroxyacetophenone, which are milder and less likely to induce irritation. This formulation strategy likely contributed to the absence of adverse reactions, such as burning or tingling, as reported by all participants in their self‐evaluations. Furthermore, individuals with compromised skin barriers may benefit from the mask's strong skin‐repairing properties.

Despite these promising results, our study had several limitations. The clinical trial employed a single‐blind, before and after design rather than a split‐face comparison, which may introduce bias in evaluating the outcomes. Moreover, the relatively small sample size limits the generalizability of the findings. Future research should include a larger cohort and utilize a double‐blind design to further validate the efficacy of the multi‐component facial mask.

## 5 Conclusion

In summary, our study provides clinical evidence supporting the efficacy of the newly designed multi‐component facial mask in enhancing skin rejuvenation and anti‐aging effects. A single use of the facial mask significantly enhances skin hydration, soothes the skin, and improves skin barrier function. Furthermore, it also reduces redness, improves skin elasticity, firmness, and texture, while diminishing the appearance of wrinkles. Long‐term use of the mask provides even more pronounced benefits. These findings highlight the potential of facial mask products in addressing skin aging and enhancing overall skin health.

## Author Contributions

Fan Yang and Hua Wang conceived, coordinated, and designed the study. Miao Guo carried out the data collection. Jinjin Zhu and Hua Wang analyzed the data and wrote the paper. All authors reviewed the results and approved the final version of the manuscript.

## Ethics Statement

This study was approved by the Shanghai Ethics Committee for Clinical Research (No. SHA01‐24070419‐JC‐01). All participants provided signed informed consent before enrollment.

## Conflicts of Interest

The authors declare no conflicts of interest.

## Data Availability

The data that support the findings of this study are available from the corresponding author upon reasonable request.
